# Comprehensive Analysis of Potential ceRNA Network and Different Degrees of Immune Cell Infiltration in Acute Respiratory Distress Syndrome

**DOI:** 10.3389/fgene.2022.895629

**Published:** 2022-06-01

**Authors:** Jiaxin Hu, Shanhui Ge, Borui Sun, Jianwei Ren, Jiang Xie, Guangfa Zhu

**Affiliations:** Department of Respiratory and Critical Care Medicine, Beijing Anzhen Hospital, Capital Medical University, Beijing, China

**Keywords:** acute respiratory distress syndrome—ARDS, sepsis, immune infiltration, competitive endogenous RNA (ceRNA) network, bioinformatic analysis

## Abstract

Acute respiratory distress syndrome (ARDS) is a leading cause of death in critically ill patients due to hypoxemic respiratory failure. The specific pathogenesis underlying ARDS has not been fully elucidated. In this study, we constructed a triple regulatory network involving competing endogenous RNA (ceRNA) to investigate the potential mechanism of ARDS and evaluated the immune cell infiltration patterns in ARDS patients. Overall, we downloaded three microarray datasets that included 60 patients with sepsis-induced ARDS and 79 patients with sepsis alone from the public Gene Expression Omnibus (GEO) database and identified differentially expressed genes (DEGs, including 9 DElncRNAs, 9 DEmiRNAs, and 269 DEmRNAs) by R software. The DEGs were subjected to the Gene Ontology (GO) and Kyoto Encyclopedia of Genes and Genomes (KEGG) for functional enrichment analysis, and a protein–protein interaction (PPI) network was generated for uncovering interactive relationships among DEmRNAs. Then, a ceRNA network that contained 5 DElncRNAs, 7 DEmiRNAs, and 71 DEmRNAs was established according to the overlapping genes in both DEGs and predicted genes by public databases. Finally, we identified the TUG1/miR-140-5p/NFE2L2 pathway as the hub pathway in the whole network through Cytoscape. In addition, we evaluated the distribution of 22 subtypes of immune cells and recognized three differentially expressed immune cells in patients with sepsis-induced ARDS by “Cell Type Identification by Estimating Relative Subsets of Known RNA Transcripts (CIBERSORT)” algorithm, namely, naive B cells, regulatory T cells, and eosinophils. Correlations between differentially expressed immune cells and hub genes in the ceRNA network were also performed. In conclusion, we demonstrated a new potential regulatory mechanism underlying ARDS (the TUG1/miR-140-5p/NFE2L2 ceRNA regulatory pathway), which may help in further exploring the pathogenesis of ARDS.

## Introduction

Acute respiratory distress syndrome (ARDS) is a fatal clinical syndrome characterized by an influx of proinflammatory leukocytes, protein-rich lung edema, and refractory hypoxemia (Sweeney and McAuley, 2016). ARDS results from multiple risk factors, among which pneumonia, sepsis, and aspiration account for 85% of all causes (Thompson et al., 2017). Whatever be the cause of ARDS, it is widely recognized that uncontrolled and exaggerated inflammatory and host-defense immune response are central to the pathogenesis underlying ARDS. (Matthay et al., 2019). Although the mortality of ARDS has decreased significantly with the advances in mechanical ventilation and symptomatic treatment, it still remains as high as 25–40%, and no precise treatments or specific biomarkers are available for the critically ill patients (Bellani et al., 2016; Pham and Rubenfeld, 2017). Furthermore, the worldwide pandemic of coronavirus disease 2019 (COVID-19) has also presented an urgent need for exploring the specific mechanism of ARDS and identifying promising biomarkers.

Long noncoding RNAs (lncRNAs) are defined as RNAs longer than 200 nucleotides that have no ability to encode proteins (Kopp and Mendell, 2018). Importantly, lncRNAs perform regulatory functions at multiple levels, including gene transcription, translation, and epigenetics (Statello et al., 2021). Recently, it has been proposed that lncRNA dysregulation is closely related to the pathophysiological process of ARDS (Chen et al., 2020, Chen et al., 2021). For instance, Nan et al., (2020) found that lncRNA MALAT1 was upregulated in lipopolysaccharide (LPS)-induced acute lung injury (ALI) and promoted apoptosis and inhibited the viability of pulmonary alveolar epithelial cells. Therefore, specific lncRNA biomarkers are considered being associated with the diagnosis and prognosis of ARDS.

MicroRNAs (miRNAs) are defined as a subtype of noncoding, single-stranded RNAs containing 19–24 nucleotides (Chen et al., 2019). MiRNAs can hybridize the 3′untranslated regions (UTRs) of target mRNAs through seed sequences, which subsequently leads to the degradation or translation inhibition of the target mRNAs (Fabian and Sonenberg, 2012). Approximately 30% of all genes are regulated by miRNAs (Krol et al., 2010), and accumulating evidence has revealed that miRNAs are associated with ARDS pathogenesis (Martucci et al., 2020; Parzibut et al., 2021). For example, Liu et al., (2017) found that the expression level of miR-200c-3p was increased in the pneumonia-induced ALI/ARDS mouse model and that miR-200c-3p overexpression led to angiotensin II upregulation, thereby aggravating lung injury *via* inhibiting the ACE2 levels. Zheng et al., (2018) found that plasma miR-27b and miR-221 levels were distinctly lower in extrapulmonary ARDS (ARDSexp) patients compared with pulmonary ARDS (ARDSp) patients and that the plasma levels of miR-26a and miR-27a were significantly lower in nonsurvival patients than survival patients in the ARDSp group. Salmena et al., (2011) proposed an innovative hypothesis, namely, the competing endogenous RNA (ceRNA) mechanism, which indicates that lncRNAs could serve as endogenous molecules to sponge target miRNAs and indirectly regulate the levels of downstream mRNAs in the cytoplasm. The ceRNA network establishes a link between ncRNAs and protein-coding mRNAs. It has been reported that the ceRNA network actively participates in the pathogenesis of respiratory diseases, such as chronic obstructive pulmonary disease (COPD), asthma, and lung malignancies (Qiu et al., 2019; Shen et al., 2020; Wu et al., 2020). Nevertheless, there are few studies about the ceRNA regulatory network in ARDS.

Importantly, the initiation and exacerbation of ARDS involves a complicated network that entails numerous molecules and various pathways, including autoimmunity, inflammation, vascular injury, and fibrosis, among which immune cells (such as T cells, macrophages, and eosinophils) have attracted substantial attention (Kumar, 2020). For instance, Mock et al., (2014) found that the number of Foxp3+ regulatory T cells (Tregs) peaked at 7 days after ALI in the mouse model and enhanced the proliferation of alveolar epithelial cells and lung regeneration. Li et al., (2019) suggested that the aggregation of classical dendritic cells in the lung promoted acute inflammation in the LPS-induced ALI model through increasing neutrophil infiltration and Th1-skewed cytokine generation. However, few studies of ARDS have comprehensively evaluated the infiltration levels of different kinds of immune cells.

Because of the extensive applications of high-throughput RNA sequencing technology, the development of bioinformatics has also achieved great progress. Many researchers work on combining bioinformatics analysis with sequencing data for further exploring the pathogenesis of human disease by noninvasive, sensitive, and accurate methods (Zhang et al., 2021a; Zhang et al., 2021b; Liu et al., 2022). For instance, Luo et al., (2021a) found that lncRNA4344 was upregulated in the LPS-induced ALI and could sponge miR-138- 5p to promote pyroptosis. Liu et al., (2021) constructed a novel hsa-miR-663b-related ceRNA network targeting autophagy in the LPS-induced ALI according to the transcriptome sequencing data.

In this work, we aimed to construct a ceRNA network related to ARDS by the bioinformatic methods. First, we downloaded the three microarray datasets about sepsis-induced ARDS from the public Gene Expression Omnibus (GEO) database and identified the differentially expressed genes (DEGs, including DElncRNAs, DEmiRNAs, and DEmRNAs) between the patients with sepsis-induced ARDS and controls. Functional enrichment analysis was performed to explore the potential function roles and pathways. Then, a key ceRNA network was established based on the overlapping genes between the DEGs and genes predicted by the bioinformatic database, and a hub pathway was recognized according to the correlation analysis and Cytoscape. Moreover, the “Cell Type Identification by Estimating Relative Subsets of Known RNA Transcripts” (CIBERSORT) algorithm was utilized to evaluate the immune cell infiltration between the patients with sepsis-induced ARDS and patients with sepsis alone.

## Materials and Methods

### Microarray Data Collection and Analysis

The three microarray datasets (GSE10474 (Howrylak et al., 2009), GSE32707 (Dolinay et al., 2012), and GSE66890 (Kangelaris et al., 2015)) were downloaded from the GEO database. To minimize data bias, we selected the sepsis-induced ARDS (one of the most common types of ARDS) as the disease group, and patients with sepsis alone (not healthy individuals) as the control groups. The GSE10474, GSE32707, and GSE66890 were detected by GPL571, GPL10558, and GPL GPL6244, respectively.

Each initial dataset profile was matched with the corresponding clinical information, and the Gene ID number transformation was conducted according to the platform annotation and GENECODE database. All microarray data were log_2_ transformed and normalized for further analysis. Repeated gene symbol value in the matrix was averaged. Next, the three expression matrices were merged into one dataset, and the batchtype effect caused by the external experimental conditions was eliminated using the “sva” package of R software (version 4.1.1) (Leek et al., 2012).

### DEG Analysis

The DElncRNAs, DEmiRNAs, and DEmRNAs in patients with the sepsis-induced ARDS were screened by the “limma” package of R software (Ritchie et al., 2015). We set the cutoff point of |log2 fold change (FC)| >0.1 and *p.*value < 0.05 for DElncRNAs, |logFC| >0.08 and *p.*value < 0.05 for DEmiRNAs, and |logFC| >0.3 with *p.*value < 0.05 for DEmRNAs. Volcano plots and heatmap of the DEGs were visualized using the “pheatmap” and “ggplot2” package of R software (version 4.1.1).

### Functional Enrichment Analysis

For DEmRNAs, we performed the Gene Ontology (GO) and Kyoto Encyclopedia of Genes and Genomes (KEGG) pathway enrichment analysis to uncover potential molecular and pathway in ARDS by the DAVID database (Dennis et al., 2003). The results were visualized by the “ggplot2” package of R software (version 4.1.1). Regarding DEmiRNAs, we performed the GO analysis and biological pathway enrichment analysis through the FunRich software (version 3.1.3) (Pathan et al., 2015). The *p.*value <0.05 was defined as the threshold for statistical significance. Finally, we used the lncLocator database to predict the subcellular localization of DElncRNA based on their sequences (Cao et al., 2018).

### Protein–Protein Interaction Regulatory Network Analysis

The online STRING database was used to explore the interactive relationships among the DEmRNAs (Szklarczyk et al., 2019). The initial PPI network was established with the interaction scores >0.7 on STRING (high confidence). Then, the top 100 hub genes were qualified by Cytoscape according to the Matthews correlation coefficient (MCC) algorithm. The final PPI network that included the top 100 hub genes was presented by the Cytoscape software (version 3.9.0) (Chin et al., 2014).

### Establishing the ceRNA Network

According to the ceRNA hypothesis, lncRNA could regulate mRNA levels by sponging the matched miRNA, we established the ceRNA network by the following steps: (1) TargetScan 8.0 was utilized to predict the target mRNAs of the DEmiRNAs and set up the miRNA-mRNA node (Agarwal et al., 2015); (2) DIANA-LncBase (version3) database was used to predict the lncRNAs matching the DEmiRNAs with a threshold >0.8, and build the lncRNA-miRNA interaction pairs (Dennis et al., 2003); (3) Venn diagram was used to determine overlapping genes between the predicted genes (TargetScan 8.0 and DAVID databases) and DEGs are observed in microarray datasets; (4) an initial ceRNA triple regulatory network was established according to the abovementioned eligible genes, and the chart was visualized by the Cytoscape software (version 3.9.0). The top 15 hub genes were identified using the “MCC” algorithm in the Cytoscape’s cytoHubba plugin; and (5) correlation analysis was performed for the top 15 hub genes in the ceRNA network by the “corrplot” package of R software (version 4.1.1).

### Profiles of Immune Cell Infiltration

The infiltration level of 22 immune cells subtypes between ARDS patients and controls was evaluated by the “CIBERSORT” package in R software that include 22 subtypes of the immune cells and specific CIBERSORT algorithm (Newman et al., 2015). We put the three expression profiles into the R software (version4.1.1) along with “e1071”, “parallel”, and “preprocessCore” packages and source file of “Cibersort.R”. Each kind of cell performed 1,000 permutations, and differences with *p.*value <0.05 were defined as being statistically significant. Next, we evaluated the proportions of different types of immune cells between ARDS patients and controls by performing the Wilcoxon rank-sum test with SPSS software (version 25.0) and identified the differentially expressed immune cells. Furthermore, Pearson correlation analysis was conducted between the differentially expressed immune cells and core DEmRNAs. All the results were visualized by the “corrplot”, “pheatmap”, and “vioplot” packages in R software.

### Statistical Analysis

All the data in this article were calculated by R software (version 4.1.1) or the online database automatically as mentioned previously. *p.*value < 0.05 was considered statistically significant among the different groups.

## Results

### Identification of DElncRNAs, DEmiRNAs, and DEmNAs in Patients With Sepsis-Induced ARDS

**FIGURE 1:**
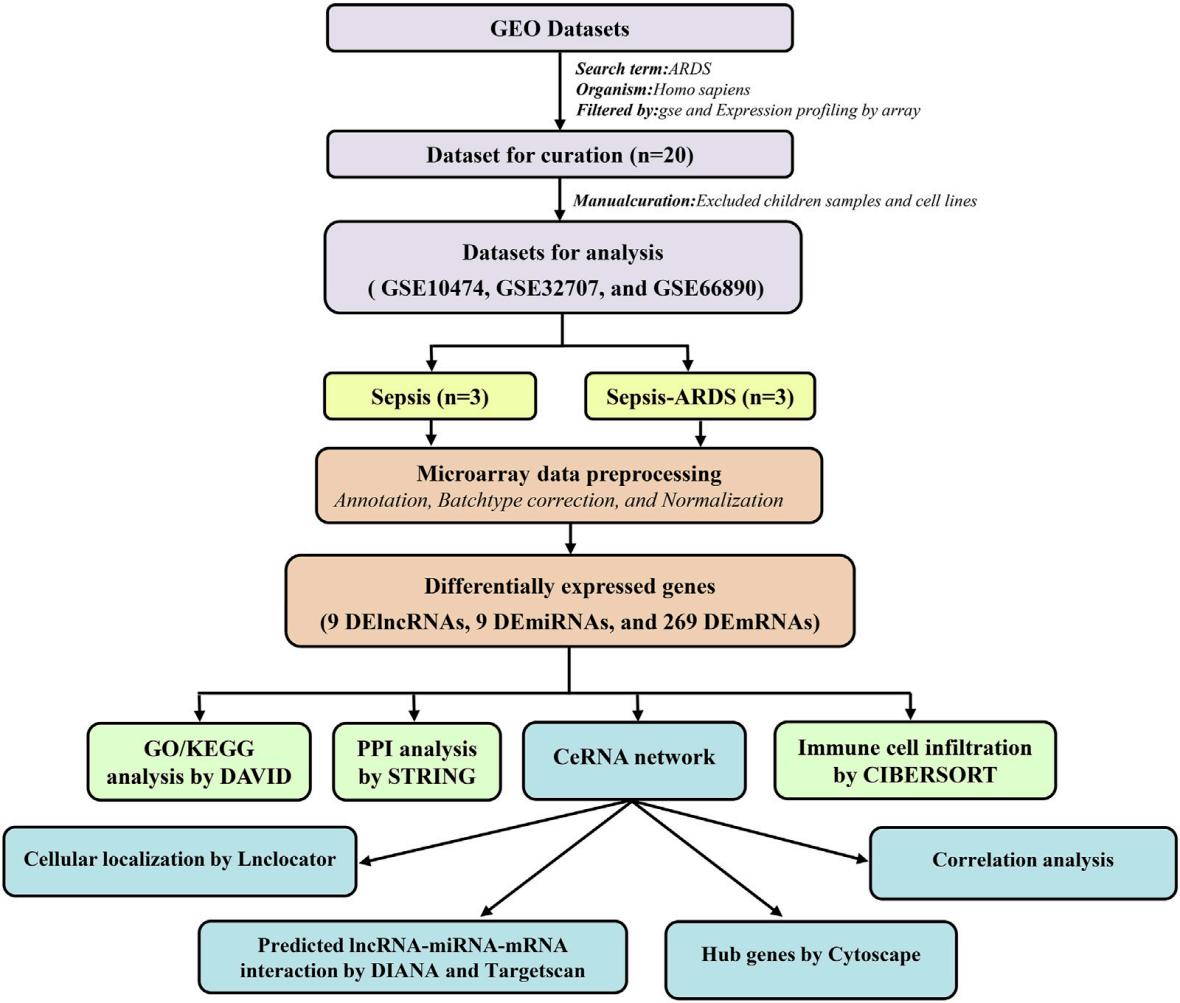
Summary of the workflow adopted in this work.

The workflow adopted in this study is illustrated in Figure 1.

**FIGURE 2:**
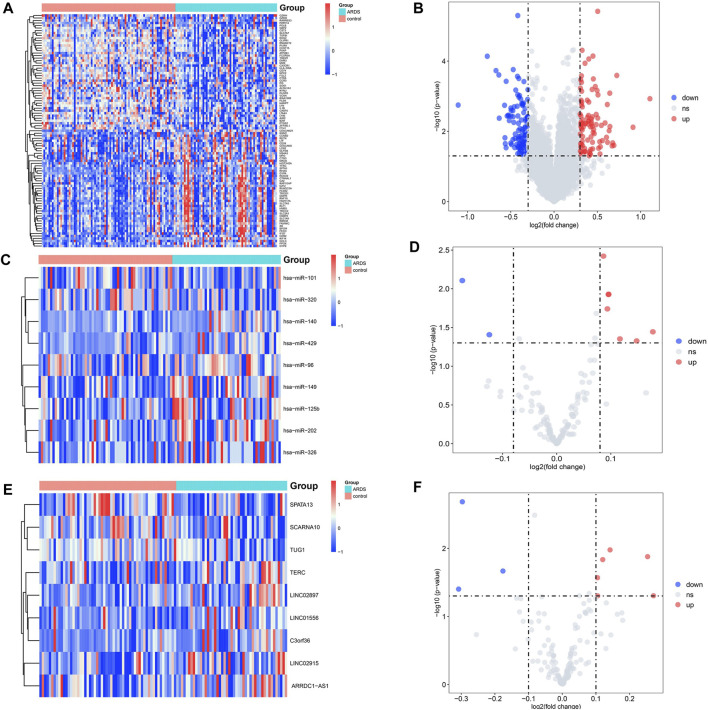
Volcano plots and heatmap of DElncRNAs, DEmiRNAs, and DEmRNAs between the patients with sepsis-induced ARDS and sepsis alone. **(A,B)** The volcano plots and heatmap for 9 DElncRNAs (|log2FC| > 0.1 and *p*.value <0.05). **(C,D)** The volcano plots and heatmap for 9 DEmiRNAs (|log2FC| > 0.08 and *p*.value <0.05). **(E,F)** The volcano plots and heatmap for the top 100 DEmRNAs (|log2fold change| > 0.3 and *p*.value < 0.05). The red dots represent the upregulated genes, and the blue dots represent the downregulated genes.

In total, three microarray datasets that contained 60 patients with the sepsis-induced ARDS and 79 patients with sepsis alone were downloaded from the GEO database. All genes are shown in Supplementary Table S1, and the details of the three microarray profiles are shown in Table 1. Because most genes in GSE10474 were mRNAs, we used GSE32707 and GSE66890 datasets for screening DElncRNAs and DEmiRNAs, respectively. In total, nine DElncRNAs (six upregulated and three downregulated) (Figures 2A,B) and nine DEmiRNAs (seven upregulated and two downregulated) (Figures 2C,D) were differentially expressed in patients with sepsis-induced ARDS compared with patients with sepsis alone. As for the dataset that merged GSE10474, GSE32707, and GSE66890, a total of 269 DEmRNAs were recognized between the sepsis-induced ARDS and controls, among which 127 genes were upregulated and 142 genes were downregulated (Figures 2E,F).

**TABLE 1:** Details of the microarray datasets used in the study.

GSE IDs	Category	Study type	Platform	Organism	Samples (n)	
					Sepsis	Sepsis-induced ARDS
GSE10474	mRNA	Expression profiling by array	GPL571	*Homo sapiens*	21	13
GSE32707	mRNA	Expression profiling by array	GPL10558	*Homo sapiens*	30	18
GSE66890	mRNA	Expression profiling by array	GPL6244	*Homo sapiens*	28	29

### Functional Enrichment Analysis of the DEmiRNAs and DEmRNAs

**FIGURE 3:**
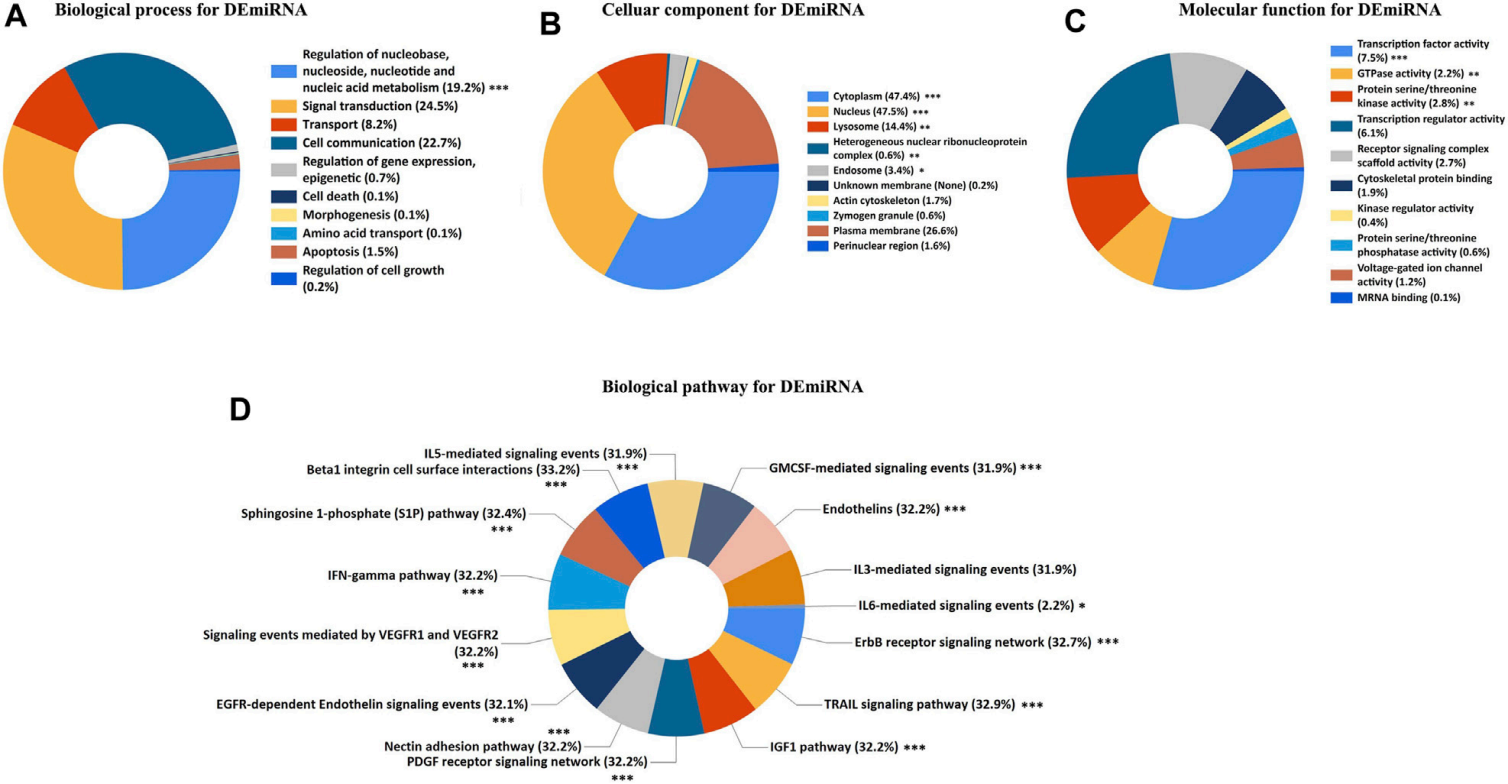
GO and biological pathway analysis for DEmiRNAs. **(A)** Biological process for DEmiRNAs. **(B)** Cellular component for DEmiRNAs. **(C)** Molecular function for DEmiRNAs. **(D)** Biological pathway for DEmiRNAs. The asterisks represented the statistical *p.*value (∗*p.*value < 0.05; ∗∗*p.*value < 0.01; ∗∗∗*p.*value < 0.001).

To explore the potential functional implications of the DEGs associated with ARDS, we performed the GO/KEGG analysis for both DEmiRNAs and DEmRNAs. Terms with a *p.*value <0.05 were considered to reflect the statistically significant differences. For the DEmiRNAs, the GO analysis demonstrated the top 10 enriched annotations for the biological processes (BPs), cellular components (CCs), and molecular functions (MFs) and the top 15 enriched annotations for the biological pathways. Specifically, the BP annotations of DEmiRNAs were mainly enriched in the regulation of nucleobase nucleosides, nucleotide, and nucleic acid metabolism (Figure 3A). The CC terms indicated that cytoplasm, nucleus, lysosome, heterogenous nuclear ribonucleoprotein complex, and endosome were the top five enriched terms (Figure 3B). Regarding the MF category, the DEmiRNAs were significantly involved in the transcription factor activity, GTPase activity, and protein serine/threonine kinase activity (Figure 3C). In terms of biological pathway enrichment analysis, the DEmiRNAs primarily converged on the IL- and G-MCSF-mediated signaling events, IFN-γ pathway, VEGFR signaling events, and EGFR-dependent endothelin signaling events, which were associated with lung inflammation and vascular injury (Figure 3D).

**FIGURE 4:**
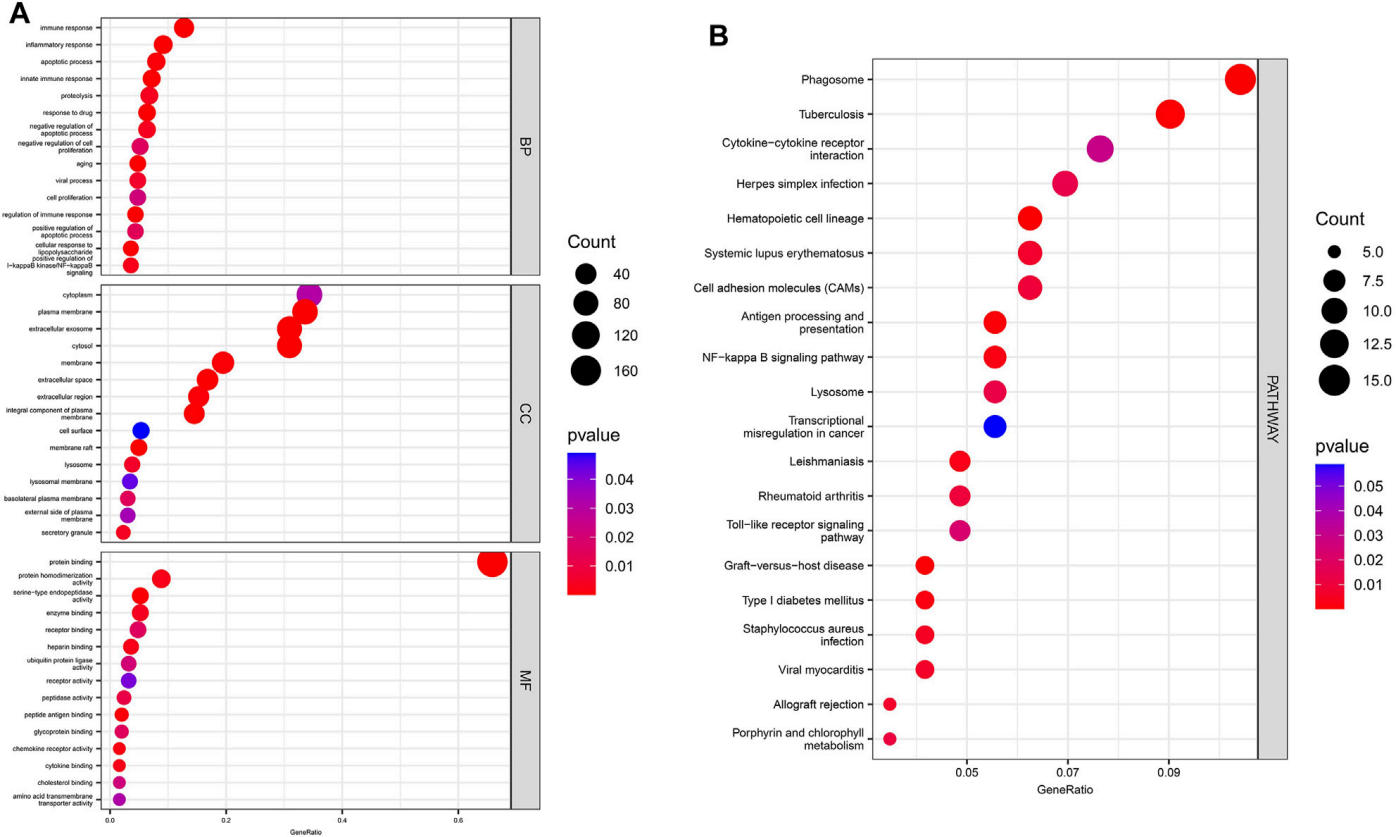
GO/ KEGG enrichment analysis for DEmRNAs. **(A)** The top 15 enriched GO terms for DEmRNAs. **(B)** The top 20 enriched terms in KEGG pathways for DEmRNAs.

The results of GO/KEGG enrichment analysis for the DEmRNAs are shown in Figure 4, In the BP annotations, the DEmRNAs were mostly associated with the following terms: innate immune response, inflammatory response, apoptotic process, and response to drug. The cytoplasm, plasma membrane, and extracellular exosome were the top three enriched terms for CCs. With respect to the MF terms, the DEmRNAs were mainly enriched in protein binding, ATP binding, protein heterodimerization activity, and serine-type endopeptidase activity. Finally, the KEGG analysis demonstrated that DEmRNAs were primarily involved in 17 pathways terms that included phagosome, infection with various pathogens (tuberculosis, herpes simplex virus, influenza A, and *Staphylococcus aureus*), cytokine−cytokine receptor interaction, antigen processing and presentation, and TLR signaling pathway.

### PPI Network Analysis

**FIGURE 5:**
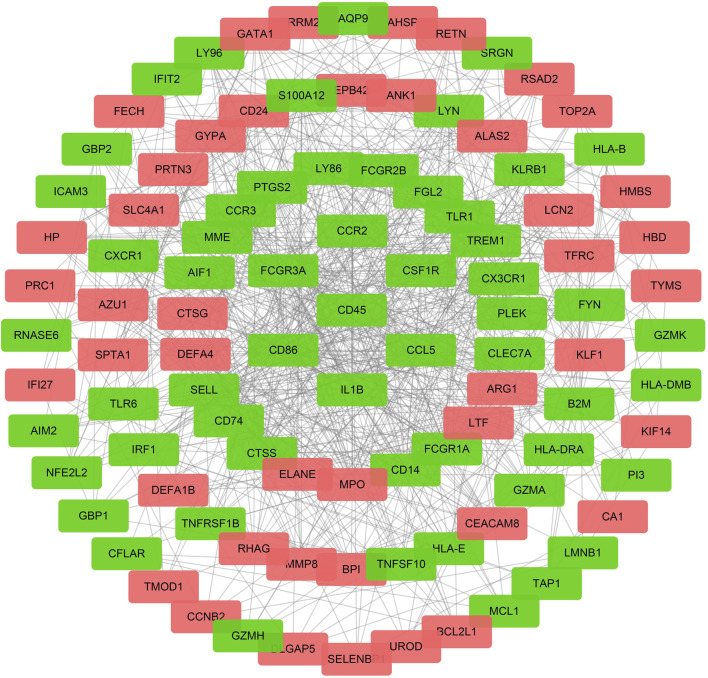
The PPI network for DEmRNAs. The red dots represent the upregulated genes, and the green dots represent the downregulated genes, the gray line represent a correlation between the two genes in DEmRNAs.

The initial PPI network containing 269 nodes and 322 edges was constructed based on the STRING online database. Next, the top 100 hub genes, which mainly included PTPRC/CD45, CD86, CD4, IL-1B, TLR2, and triggering receptor expressed on myeloid cells 1 (TREM1) and Fc gamma receptor IIIa (FCGR3A), were identified *via* cytoHubba plugin of Cytoscape’s based on the MCC algorithm. The final PPI network was constructed based on these 100 hub genes (Figure 5). These data indicated that the functions of DEmRNAs were mainly enriched in inflammation, immune response, and pathogen infection.

### Construction of the ceRNA Regulatory Network

**FIGURE 6:**
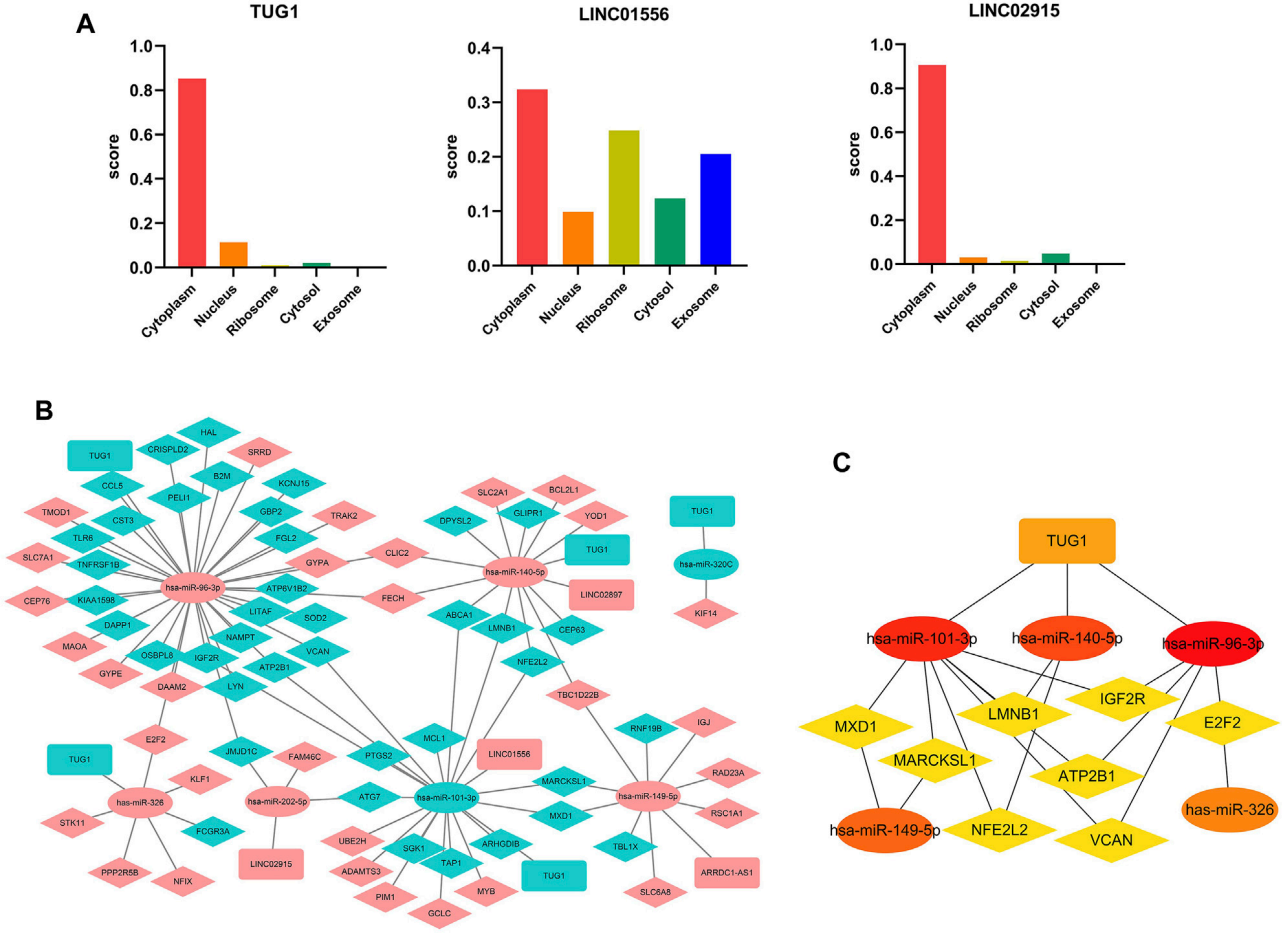
Construction of the ceRNA network **(A)** The subcellular localization for DElncRNAs predicted by lncLocator. **(B)** The triple ceRNA regulatory network in sepsis-induced ARDS. The red dot indicates upregulated genes, and the blue dot represents downregulated genes. The ellipses denote miRNAs, diamonds denote mRNAs, and round rectangles denote lncRNAs. **(C)** The hub genes in the ceRNA network calculated by MCC algorithm in Cytoscape.

Considering that the subcellular localization of lncRNAs determines their potential functions, we estimated the cellular localization of all the nine DElncRNAs *via* the lncLocator before constructing the ceRNA regulatory network. As shown in Figure 6A, LINC01556, LINC02915, and TUG1 were mainly distributed in the cytoplasm, but the other six lncRNAs (ARRDC1-AS1, LINC02897, SCARNA10, SPATA13, TERC, and C3orf36) were mainly located in the exosome or cytosol (Supplementary Figure S1). These results suggest that LINC01556, LINC02915, and TUG1 may act as a ceRNA to sponge targeting miRNA and regulate the expression level of matching mRNA.

For the initial lncRNA-miRNA-mRNA regulatory network, we obtained 5 lncRNAs in total (4 upregulated that is ARRDC1-AS1, LINC02897, LINC01556, and LINC02915, and 1 downregulated that is TUG1), 7 miRNAs (2 downregulated include hsa-miR-101-3p and hsa-miR-320c, 5 upregulated include hsa-miR-96-3p, hsa-miR-140-5p, hsa-miR-149-5p, has-miR-202-5p, and hsa-miR-326), and 71 mRNAs (31 upregulated and 40 downregulated) by selecting the overlapping genes between the predicted genes (TargetScan 8.0 and DIANA-LncBase databases) and DEGs in our microarray datasets, the results were visualized by the Cytoscape software **(** Figure 6B). Subsequently, we used the “MCC” algorithm of the Cytoscape plugin, cytoHubba, to determine the top 15 hub genes in the initial regulatory network and construct the central ceRNA network that contained 15 nodes and 19 edges (Figure 6C). The results showed that one lncRNA (TUG1), five miRNAs (hsa-miR-96-3p, hsa-miR-101-3p, hsa-miR-140-5p, hsa-miR-149-5p, and hsa-miR-326), and eight mRNAs (ATP2B1, E2F2, IGF2R, LMNB1, MARCKSL1, MXD1, NFE2L2, and VCAN) acted as the hub genes in the whole ceRNA network.

**FIGURE 7:**
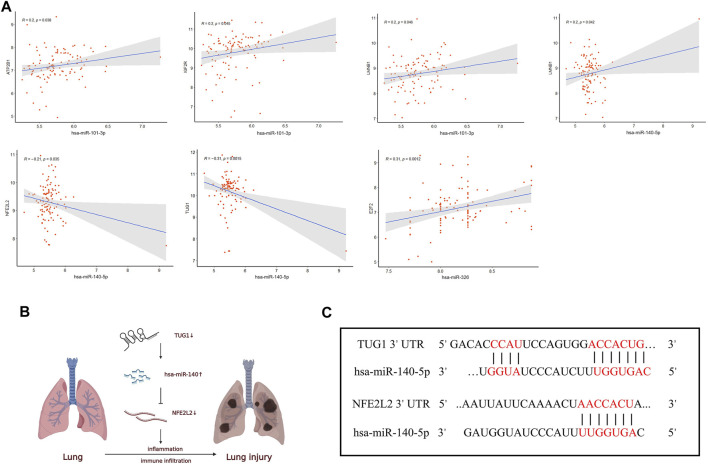
Correlation analysis of the ceRNA network. **(A)** The correlation analysis among the hub genes in the ceRNA network. **(B)** Schematic model of the ceRNA network. **(C)** Binding sites of miR-140-5p to the 3′ UTRs region of TUG1 and NFE2L2 predicted by TargetScan and DIANA.

Furthermore, Pearson correlation analysis was used to estimate the correlation of hub genes in the ceRNA network, and *p.*value <0.05 was considered statistically significant (Figure 7A). For the lncRNA-miRNA node, there existed a negative relation between TUG1 and hsa-miR-140-5p (*R* = −0.31, *p* = 0.0015). As for the miRNA-mRNA node, hsa-miR-101-3p was positively related with ATP2B1 (R = 0.2, *p* = 0.038), IGF2R (R = 0.2, *p* = 0.045), and LMNB1 (R = 0.2, *p* = 0.046). hsa-miR-140-5p was negatively related with NFE2L2 (*R* = -0.21, *p* = 0.035) and positively related with LMNB1 (R = 0.2, *p* = 0.042). hsa-miR-326 was positively related with E2F2 (*R* = 0.31, *p* = 0.0012). The remaining correlation results which were nonsignificant are shown in Supplementary Figure S2.

Therefore, UG1 may serve as a ceRNA to upregulate the expression of NFE2L2 through sponging miR-140-5p according to the ceRNA hypothesis (Figure 7B). The binding sites of hsa-miR-140-5p to the 3′ UTRs region of TUG1 and NFE2L2 were predicted by DIANA and TargetScan (Figure 7C), respectively. Thus, the TUG1/miR-140-5p/NFE2L2 ceRNA network model was constructed.

### Analysis of Infiltrating Immune Cells in Sepsis-Induced ARDS

**FIGURE 8:**
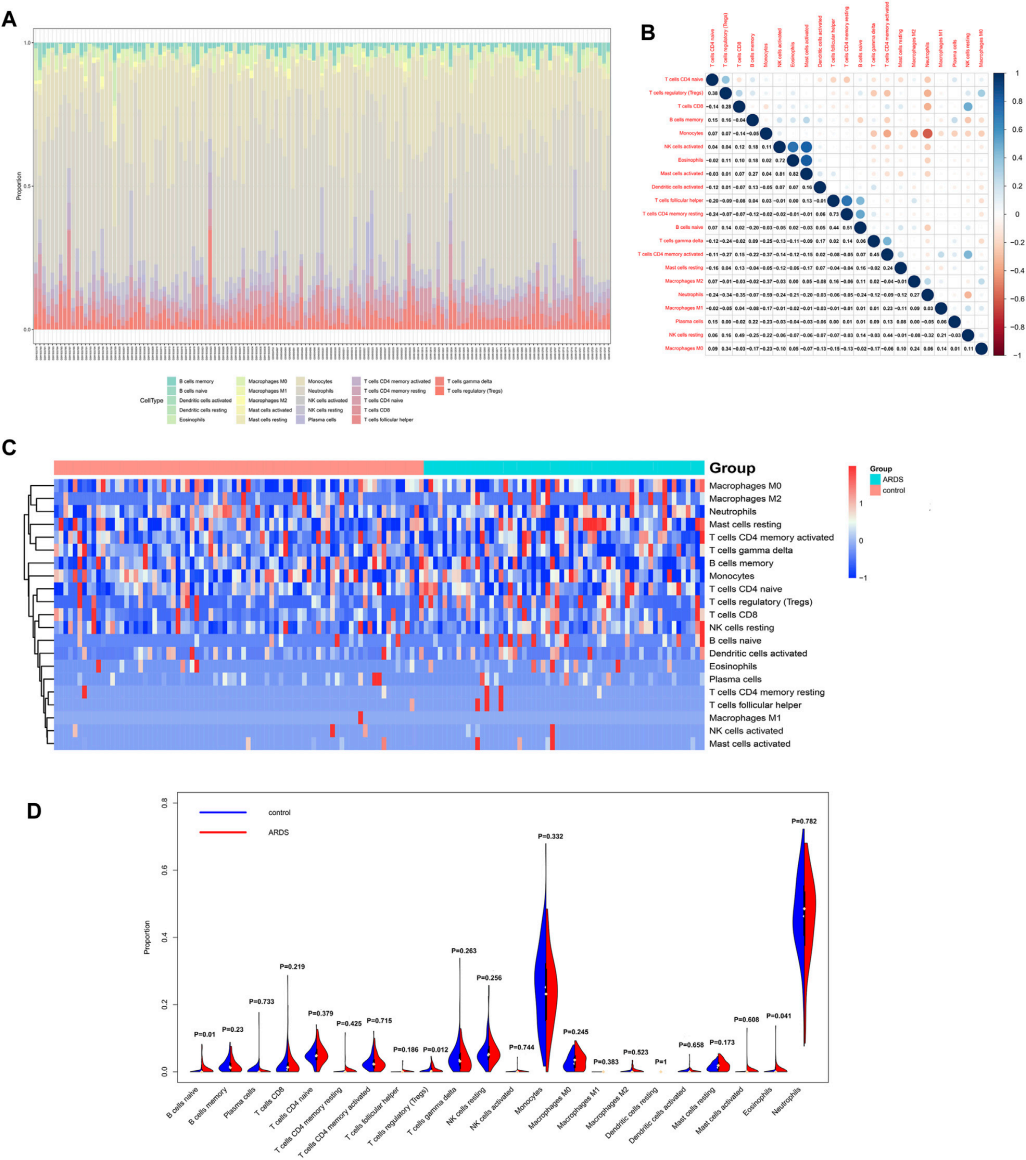
Analysis of 22 subtypes of immune cells in patients with sepsis-induced ARDS. **(A)** The distribution of immune cell types in total 139 samples. **(B)** The correlation analysis among 22 immune cell subtypes. **(C)** The heatmap for infiltrating immune cells. **(D)** The violin plots for infiltrating immune cells.

The distributions of 22 subtypes of infiltrating immune cells in patients with sepsis-induced ARDS and sepsis alone were calculated by the CIBERSORT algorithm in R software (Figure 8A). The results showed that neutrophils and monocytes contributed to the two most abundant parts. The correlation among all the 22 immune cells subtypes were estimated, and the results were illustrated in Figure 8B. Specifically, eosinophils was positively related with the activated natural killer (NK) cells (*R* = 0.72) and activated mast cells (*R* = 0.82); activated natural killer (NK) cells was positively related with activated mast cells (R = 0.81); memory CD4^+^ T cells were positively associated with the follicular T helper cells (*R* = 0.73) and naive B cells (*R* = 0.51), whereas monocytes were negatively correlated with neutrophils (*R* = -0.59). The remaining immune cell subtypes showed a weak correlation (*R*<0.5).

The infiltration levels of all immune cells subtypes between the ARDS patients and controls were evaluated by Wilcoxon rank-sum test, and the results indicated that three subtypes showed differential infiltration, that is naive B cells, Tregs, and eosinophils. Specifically, naive B cells, Tregs, and eosinophils were upregulated in ARDS patients (Figures 8C,D).

### Co-Expression Analysis Between Immune Cells and ceRNA Network

**FIGURE 9:**
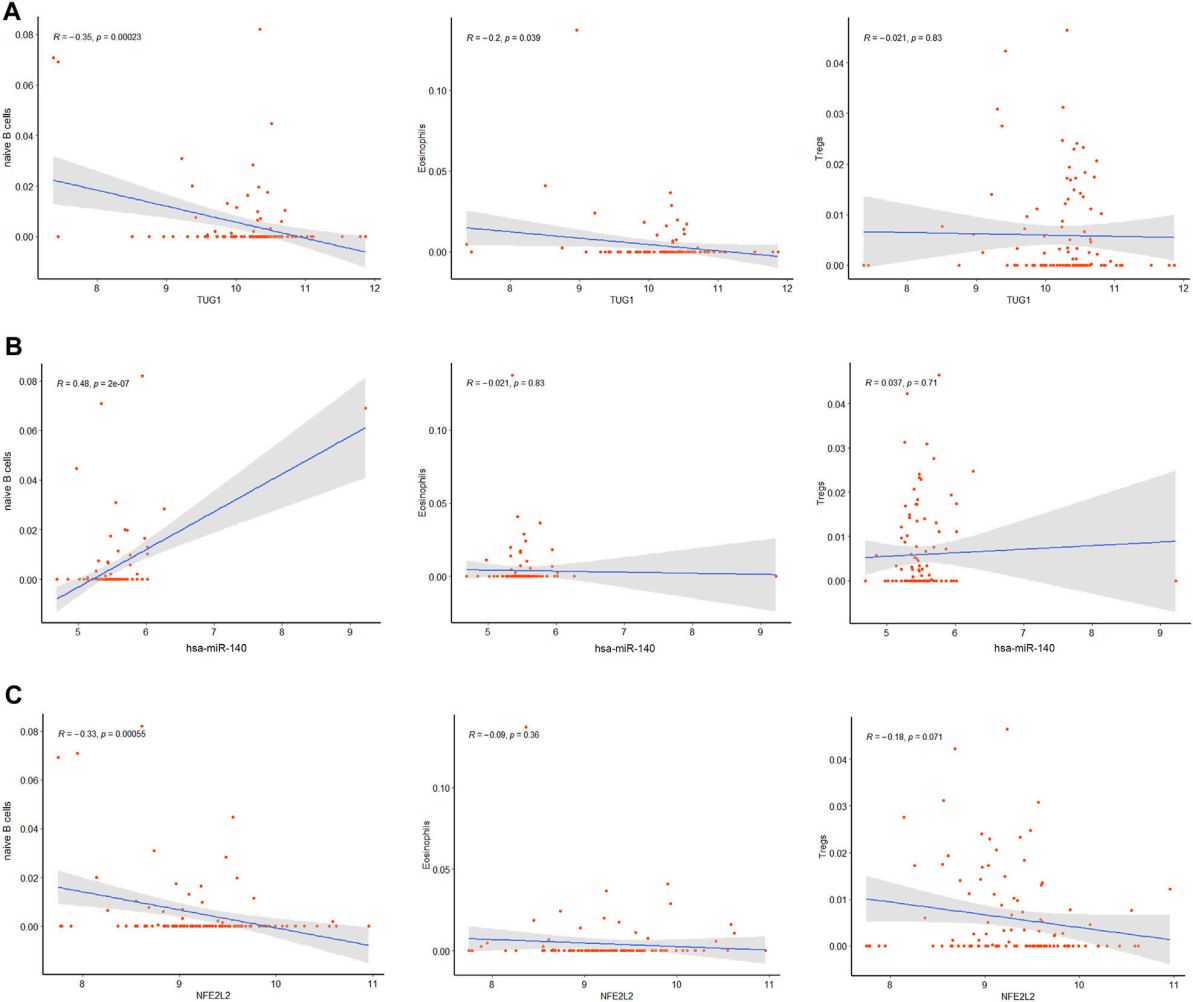
Co-expression patterns of the differentially expressed immune cells and core DEmRNAs. **(A)**The correlation between TUG1 and differentially expressed immune cells. **(B)** The correlation between miR-140 and differentially expressed immune cells. **(C)** The correlation between NFE2L2 and differentially expressed immune cells.

The correlation between the hub genes in the ceRNA network and differentially expressed immune cells was calculated by Pearson correlation analysis (Figure 9). TUG1 was negatively related with the naive B cells (*R =* −0.35, *p* = 0.00023) and eosinophils (*R =* −0.2, *p* = 0.039). hsa-miR-140-5p was positively related with the naive B cells (*R =* 0.48, *p* = 2e-07). NFE2L2 was negatively related with the naive B cells (*R =* −0.33, *p* = 0.00055). The associations between other immune cell subpopulations and genes in the ceRNA network were considered nonsignificant. These results suggest that the TUG1/miR-140-5p/NFE2L2 axis may influence the ARDS-associated immune cells infiltration.

## Discussion

ARDS, which is characterized by hypoxemic respiratory failure, is one of the most common causes of mortality and morbidity in the critically ill patients with a mortality of more than 40% (Bellani et al., 2016). Multiple causes have been confirmed to be associated with ARDS, including intra- or extrapulmonary risk factors. For any cause, uncontrolled inflammation and dysregulated immune system are considered to be the central pathogenesis of ARDS (Thompson et al., 2017). Although researchers and clinicians have achieved substantial progress in the area of pathogenesis and treatments of ARDS over the past decade (Henderson et al., 2017; Sahetya et al., 2017), there still existed many uncertainties to be elucidated. Therefore, it is necessary to investigate the potential pathological mechanisms and recognized effective biomarkers to aid diagnosis and treatment of ARDS. Following the emergence of the ceRNA hypothesis in recent years, increasing evidence has indicated that lncRNAs can act as sponges to regulate the target gene expression in ARDS by competitive binding for target miRNAs. For instance, Luo et al., (2021b) suggested that lncRNA NLRP3 could act as a ceRNA to sponge miR-138-5p and promote NLRP3 inflammasome-induced inflammatory response. The lncRNA NEAT1 can target toll-like receptor 4 to upregulate proinflammatory cytokines levels and aggravate LPS-induced lung injury both *in vitro* and *in vivo* by sponging miR- 98-5p (Chen J. et al., 2021).

In this study, we aimed to construct a ceRNA regulatory network by the bioinformatic methods to explore potential mechanism associated with ARDS. We downloaded three datasets for sepsis-induced ARDS from the GEO database and identified 9 DElncRNAs (ARRDC1-AS1, C3orf36, SCARNA10, SPATA13, TERC, TUG1, LINC01556, LINC02897, and LINC02915), 9 DEmiRNAs (hsa-miR-96, hsa-miR-101, hsa-miR-125b, hsa-miR-140, hsa-miR-149, hsa-miR-202, hsa-miR-320c, hsa-miR-326, and hsa-miR-429), and 269 DEmRNAs. Recent findings have confirmed that some of these DElncRNAs and DEmiRNAs are indeed involved in lung disease. For example, the lncRNA TERC plays an important role in maintaining the telomere length. It was previously confirmed that TERC was amplified in non-small-cell lung carcinoma, and TERC based therapies have the potential function to promote apoptosis of the cancer cells with a high specificity (Gala and Khattar, 2021). Huang et al., (2017) found that miR-101 was downregulated in the lungs of patients with idiopathic pulmonary fibrosis (IPF), and overexpression of miR-101 in the mouse lungs ameliorated bleomycin-induced lung fibrosis by suppressing the TGF-β-induced activation and WNT5a-induced proliferation of lung fibroblasts. Additionally, our functional enrichment analysis demonstrated that DEmiRNAs were primarily involved in some widely recognized signaling pathways that are associated with acute inflammatory response and regulation of epithelial–endothelial barrier, such as IL-mediated signaling pathways, the IFN-γ pathway, VEGFR signaling events, and EGFR-dependent endothelin signaling events, indicating that our results were credible.

Next, we analyzed the DEmRNAs further by performing the GO/KEGG annotation and constructing PPI network in sepsis-induced ARDS. We found that whether the enriched GO/KEGG terms or hub genes in the PPI network, the related DEmRNAs primarily converged on terms associated with immune and inflammation responses, including cell apoptosis, cell phagosome, and various pathogen-related infection. CD45, CD4, and CD86 were the classical cell markers of neutrophils, T cells, and B cells, and TNF, IL, and TLR have been widely recognized crucial for the pathogenesis of ARDS (Butt et al., 2016). AIM2 is a kind of cytosolic pattern recognition receptors that can initiate the assembly of the inflammasome, and promote the generation of inflammatory cytokines, such as IL-1β and IL-18. AIM2 activation can also trigger pyroptosis, a proinflammatory form of cell death (Lugrin and Martinon, 2018). Li et al., (2021) found that AIM2 inflammasomes were activated in alveolar macrophages from the LPS-induced ARDS mouse model *via* NET pathway and lead to caspase-1-dependent pyroptosis. Cho et al., (2020) revealed that AIM2 inflammasome activation exacerbate lung fibrogenesis during bacterial infection *via* GLUT1-mediated glycolysis. ATG7 and ATG9A were autophagy markers, and increasing evidence proves that autophagy participate in the pathological process of diverse lung diseases, including ALI, IPF, COPD, pulmonary arterial hypertension, and cystic fibrosis through regulating inflammatory response, oxidative stress balance, DNA damage and repair, apoptosis, and necroptosis in different type of cells (Wang et al., 2019; Vishnupriya et al., 2020).

Foremost, an initial triple regulatory ceRNA network was established based on DElncRNAs, DEmiRNAs, and DEmRNAs that overlapped with the genes predicted by Targetscan 8.0 and DIANA-lncBase. Furthermore, we screened out the top15 hub genes using cytoHubba in Cytoscape and performed Pearson correlation analysis to obtain the final triple regulatory ceRNA network. To sum up, a TUG1/miR-140-5p/NFE2L2 axis associated with the sepsis-induced ARDS was established. By querying these three hub genes in PubMed, we found that TUG1, miR-140-5p, and NFE2L2 have been confirmed to be involved in various types of disease that are mainly associated with inflammation, oxidative stress, and immune system dysfunction. For instance, overexpression of TUG1 could alleviate pulmonary injury in the ALI mouse model, including inhibiting apoptosis and inflammatory response of the epithelial cell, and providing protective effect for the pulmonary microvascular endothelial cells against the LPS-induced damage (Qiu et al., 2020). Long et al., (2016) proposed that overexpression of TUG1 was associated with the improvement of mitochondrial bioenergetics in the podocytes of diabetic mice by binding to the promoter sequence of PGC-1α Fang et al., (2020) indicated that TUG1 alleviated cardiac hypertrophy both *in vivo* and *in vitro via* miR-34a/DKK1/Wnt-β-catenin signaling. For miR-140, Zhao et al., (2018) found miR-140-5p was significantly upregulated in doxorubicin-induced heart injury and promoted myocardial oxidative stress *via* inhibiting the expression of Nrf2 and Sirt2, whereas Yang et al., (2021) suggested miR-140 could suppress airway inflammation and inhibit apoptosis of the bronchial epithelial cell in asthma by targeting GSK3β. Thus, the function of miR-140 has not been fully elucidated, it may depend on the specific disease involved. NFE2L2, also known as Nrf2, was the key molecule of the keap1/Nrf2/ARE pathway, where the pathway forms the major node to protect cells and organisms against oxidative and inflammatory attack from both exogenous and endogenous origins (Hybertson et al., 2011; Yamamoto et al., 2018). It has been confirmed that Nrf2 activation plays a protective role in various diseases including autoimmune disease, chronic lung diseases, neurodegenerative diseases, metabolic disturbance, and even cancer initiation (Cuadrado et al., 2019; Lignitto et al., 2019; Saeedi et al., 2020). Crucially, the Nrf2 pathway has been found to be suppressed in patients with COVID-19 according to lung biopsies; and the pharmaceutical agonists of Nrf2 could inhibit the replication of SARS-CoV2 and corresponding inflammatory reaction (Cuadrado et al., 2020). An Nrf2 activator has obtained clinical approval and inhibitors of KEAP1 are actively being developed (Cuadrado et al., 2019). All the abovementioned data collectively indicate that TUG1, miR-140, and NFE2L2 exert a crucial effect on inflammatory response, and further exploration of this predicted ceRNA axis may provide novel aspects of the mechanism underlying ARDS.

It is worth mentioning that the pathological process in ARDS is caused by multiple factors and complicated network. In addition to inflammation cascades, dysregulation of immune system was another hallmark for ARDS. We investigated the proportion of infiltrating immune cell subtypes in the sepsis-induced ARDS patients. To the best of our knowledge, this is the first time to comprehensively estimate the infiltration levels of 22 subtypes of immune cells. We totally identified the three types of differentially expressed immune cells, that is naive B cells, Tregs, and eosinophils. B cells infiltration in airway is correlated with many respiratory diseases. Habener et al., (2021) reported that regulatory B cells could suppress the hyperreactivity and remodeling of airway and induce Tregs generation in asthma. Aziz et al., (2018) demonstrated that B-1a cells distinctly inhibited the mRNA and protein levels of IL-6, IL-1β, and MIP-2 in the sepsis-induced ALI patients and mouse model. Tregs, as an important kind of immunosuppressive cells, were found to be activated in ARDS, and the transplantation of Tregs into ARDS mice could inhibit the levels of proinflammatory cytokines and fibrocyte assembly in the lung (D'Alessio et al., 2009; Garibaldi et al., 2013). The impact of eosinophils in ARDS has gradually drawn researchers’ attention (Zhu et al., 2020), found that the number of blood eosinophils was increased in the surviving ARDS patients, independent of corticosteroid usage, and CD101^-^ eosinophils could be recruited to the alveolar space earlier than neutrophil in the LPS-initiated lung injury. Despite high levels of neutrophils and macrophages in patients with sepsis-induced ARDS in our study, we did not observe a significant difference in both cell types between ARDS patients and sepsis patients. We suspect that neutrophils and macrophages are already distinctly higher in patients with sepsis, therefore, they may not change dramatically even if ARDS occurred.

As far as we know, this is the first comprehensive analysis that combines the ceRNA regulatory network with the immune infiltration analysis in ARDS. Based on the bioinformatic methods, we identified the DEGs between patients with sepsis-induced ARDS patients and sepsis alone from the three independent microarray datasets. We further analyzed DEGs from different aspects, such as GO/KEGG and PPI analysis to screen out the genes closely related to ARDS. Finally, we established a ceRNA (TUG1/miR-140-5p/NFE2L2) network in sepsis-induced ARDS patients according to the abovementioned results. Moreover, we also evaluated the infiltration levels of differentially expressed immune cells in ARDS patients and their correlation with the core DEGs, which provide new insights into immune regulatory mechanisms in ARDS. Our results supply new prospects to explore the pathogenesis underlying ARDS and may provide potential therapeutic targets for ARDS patients, which needs to be further confirmed.

## Data Availability

The original contributions presented in the study are included in the article/Supplementary Materials, further inquiries can be directed to the corresponding author.

## Supplementary Material

Supplementary Figure S1 Cellular localization for DElncRNAs (ARRDC1-AS1, LINC02897, SCARNA10, SPATA13,TERC, and C3orf36) predicted by LncLocator.

Supplementary Figure S2 Correlation analysis among hub RNAs that have no different significance in ceRNA works.

Supplementary Table S1 The expression profiles of GSE10474, GSE32707, and GSE66890.
