# Supplementary material for: Comprehensive Analysis of Potential ceRNA Network and Different Degrees of Immune Cell Infiltration in Acute Respiratory Distress Syndrome
Source: Front Genet. 2022 Jun 1;13:895629. doi: 10.3389/fgene.2022.895629 (PMC9198558; doi:10.3389/fgene.2022.895629)
Supplement: Supplementary file 2 [file Image1.pdf]

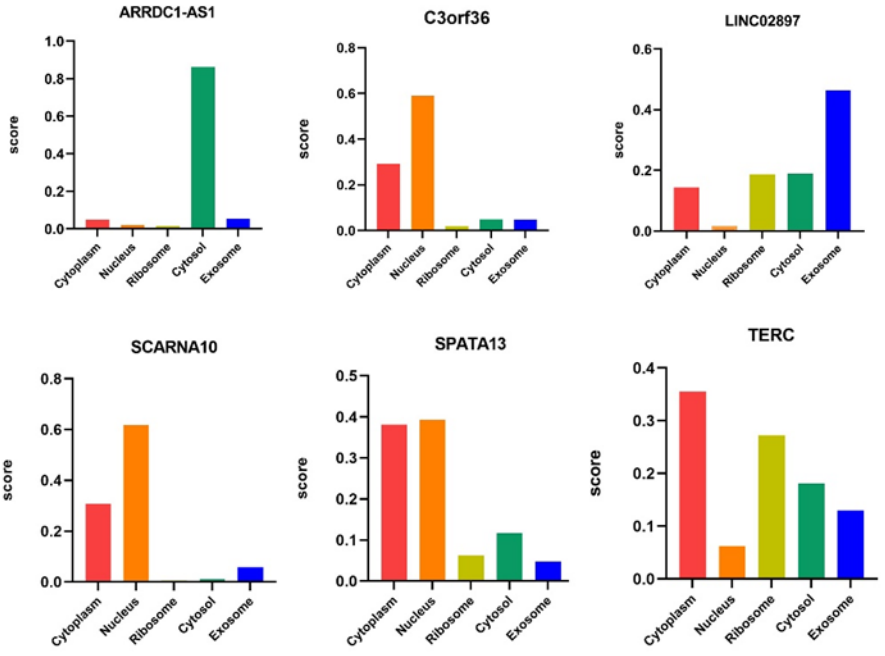

Supplementary Figure1. Cellular localization for DElncRNAs (ARRDC1-AS1, LINC02897, SCARNA10, SPATA13,TERC and C3orf36) predicted by LncLocator.

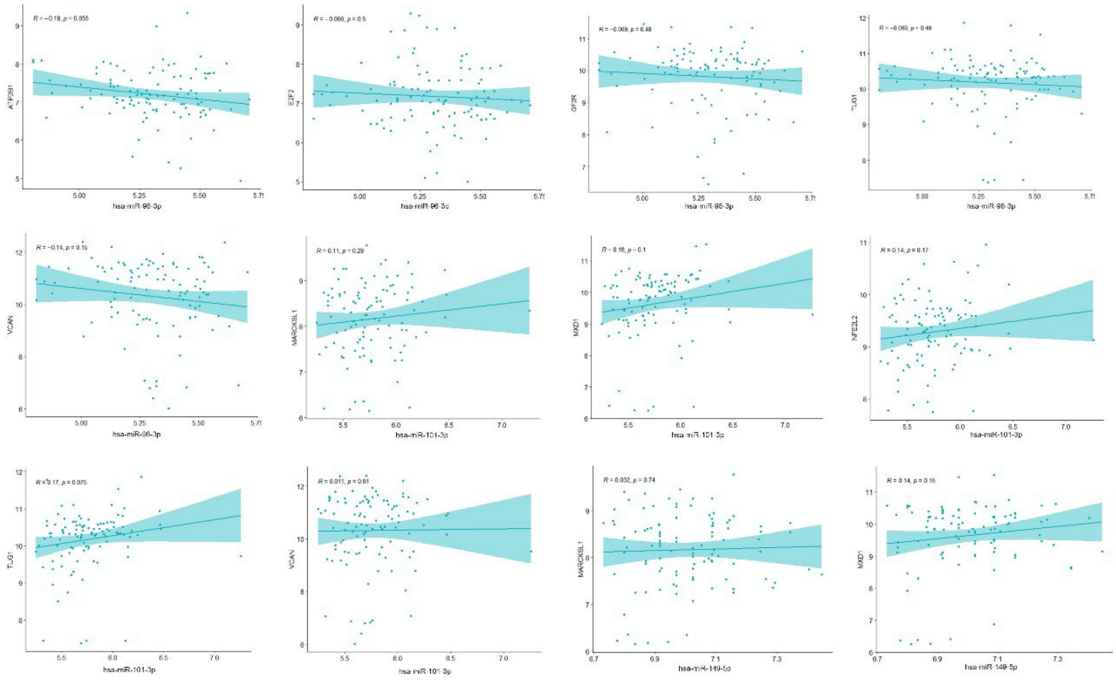

Supplementary Figure2. Correlation analysis among hub RNAs that have no different significance in ceRNA works
